# Practical Manual of Dental Casting

**Published:** 1913-07-15

**Authors:** 


PRACTICAL MANUAL OF DENTAL CASTING. This book of more than 230 pages sets forth as well as can be done the casting process as it has developed during the past three years, and been recorded in articles contributed to the *Dental Summary*. Very properly the first article is historic by L. W. Strycker, who sets forth the ancient method of casting, from which we undoubtedly got our modern conceptions of casting as applied to dental art. This is followed by about seventy-five articles on various aspects of the application of the casting process to dental plates, fillings, bridges, etc. Of course, the work could not be systematically arranged, and the object has been to present in a convenient form the material for reference. It would undoubtedly be of more service if a carefully prepared index had been incorporated in the book. It is well printed and will find a useful place in the library of every dentist. The Ransom & Randolph Co., of Toledo, O., publish the work and it can be had from any dental supply house. The price, bound in substantial cloth is $2.50.

